# Supplementary material for: Uptake of Cerium Dioxide Nanoparticles and Impact on Viability, Differentiation and Functions of Primary Trophoblast Cells from Human Placenta
Source: Nanomaterials (Basel). 2020 Jul 3;10(7):1309. doi: 10.3390/nano10071309 (PMC7407216; doi:10.3390/nano10071309)
Supplement: Supplementary file 1 [file nanomaterials-10-01309-s001.pdf]

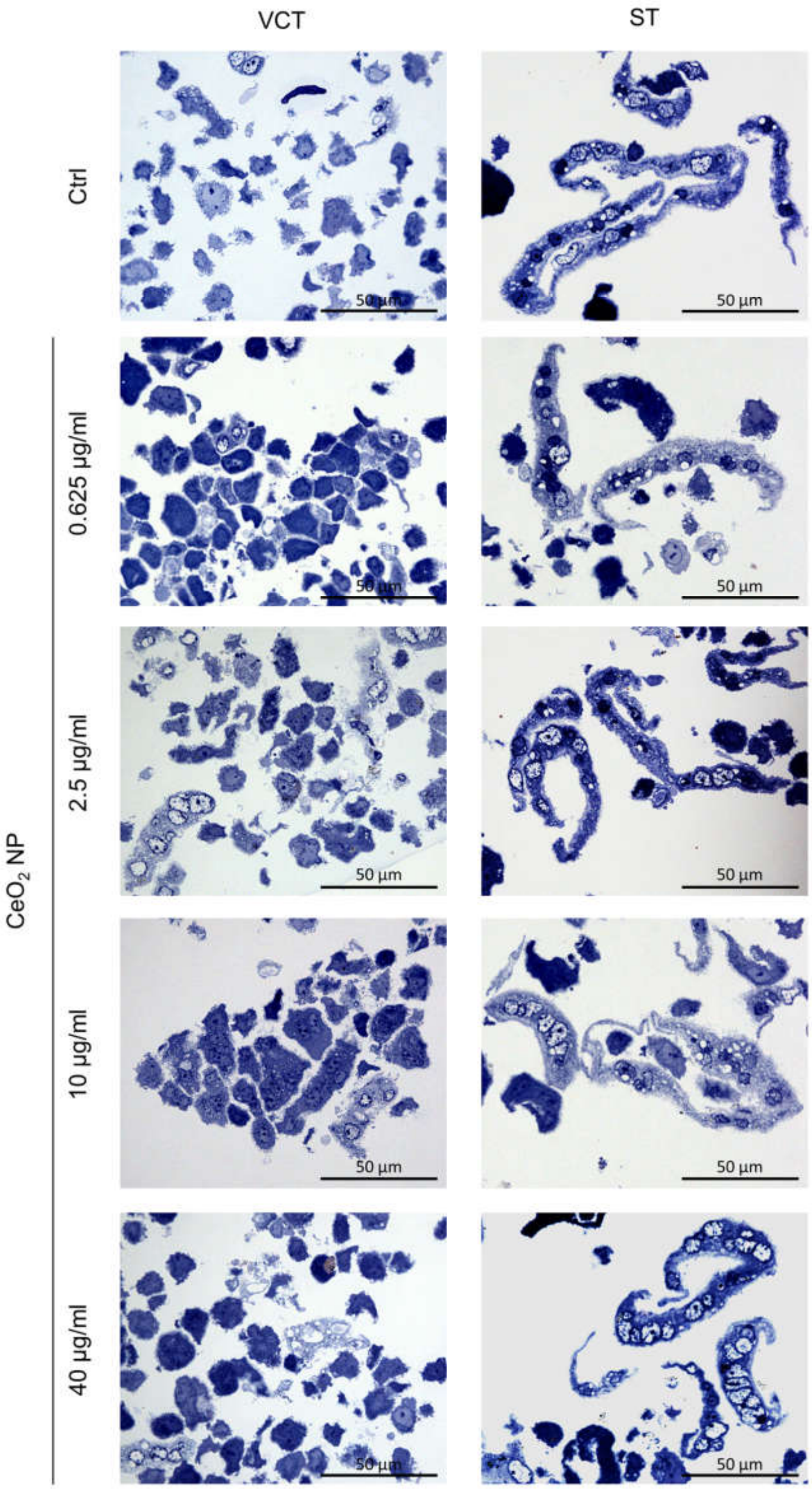

Figure S1. Toluidine blue staining of trophoblasts treated with cerium dioxide nanoparticles. VCT purified from term placenta were plated overnight and cultured for 24 h to obtain VCT or for 72 h in order to form spontaneously the syncytiotrophoblast (ST) and then were either untreated or incubated with CeO_2 NPs at the indicated concentrations for an additional 24 h, fixed, included in epoxy resin and stained with toluidine blue before transmission electron microscopy (TEM).

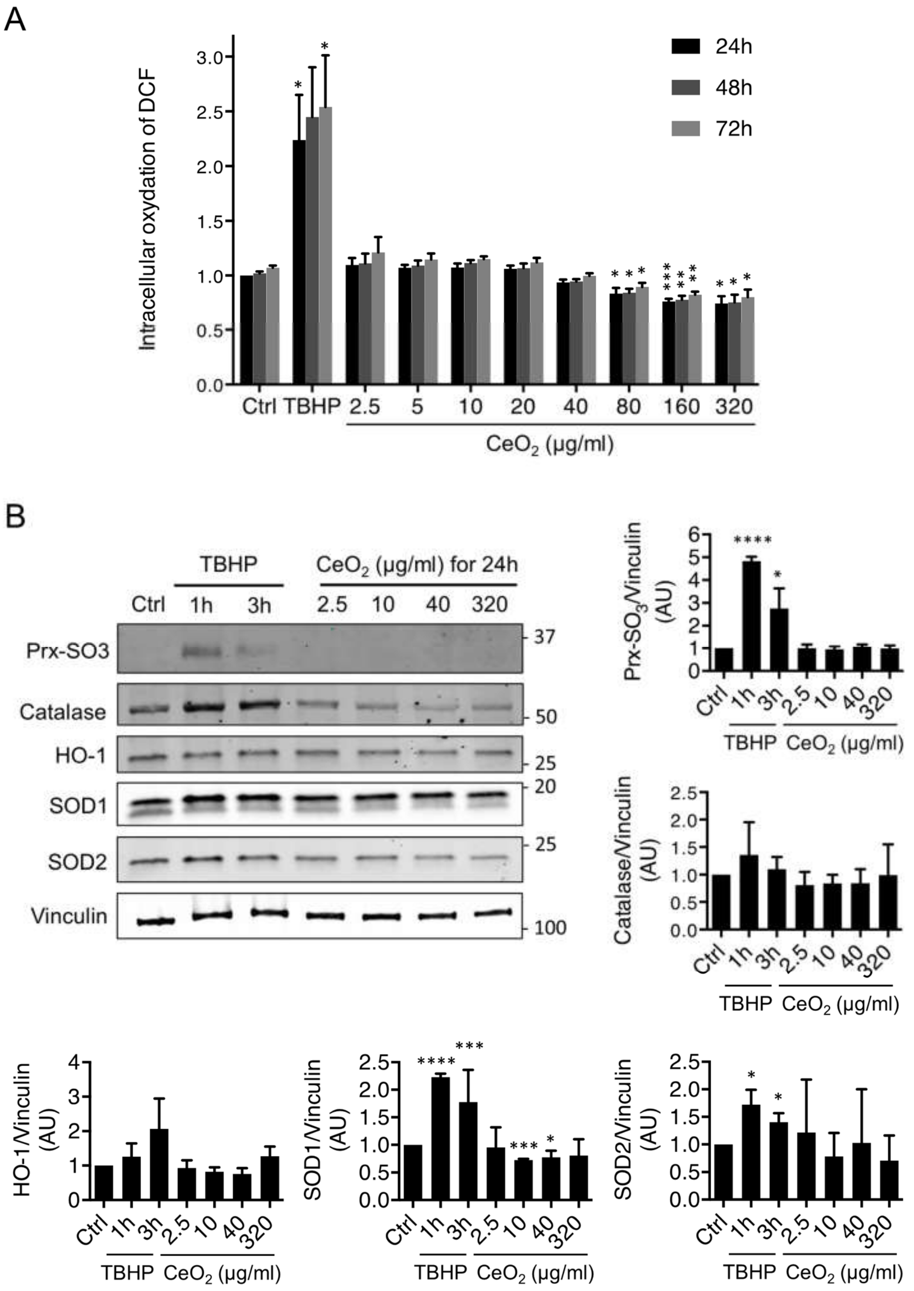

Figure S**2.** Intracellular oxidative stress after trophoblast exposure to cerium dioxide nanoparticles. VCT purified from term placenta were plated overnight and then were either untreated (Ctrl) or incubated with CeO_2 NPs at the indicated concentrations for 24 to 72 h. (**A**) ROS levels were evaluated using CM-H_2DCFDA. Tert-butyl hydroperoxide (TBHP, 250 µM) was used as positive control. All experiments were performed in triplicate and 3 independent experiments (n = 3). Data are expressed as means +/- SEM normalized to the 24 h control. *p <0.05 ; **p <0.01 ;***p <0.001. (**B**) VCT were left untreated (Ctrl), treated with tert-butyl hydroperoxide (TBHP, 100 µM) for 1 h and 3 h or with CeO_2 NPs at the indicated concentrations for 24 h of incubation. Total protein extracts were subjected to SDS-PAGE under reducing conditions and membranes were immunoblotted with anti-Prx-SO_3, anti-catalase, anti-HO1, anti-SOD1, anti-SOD2 and anti-vinculin antibodies (the latter used as loading control). Immunoblots were quantified with an Odyssey System Imager and are shown in the bar scale graph as the ratio to vinculin as mean +/- SEM (n = 3).

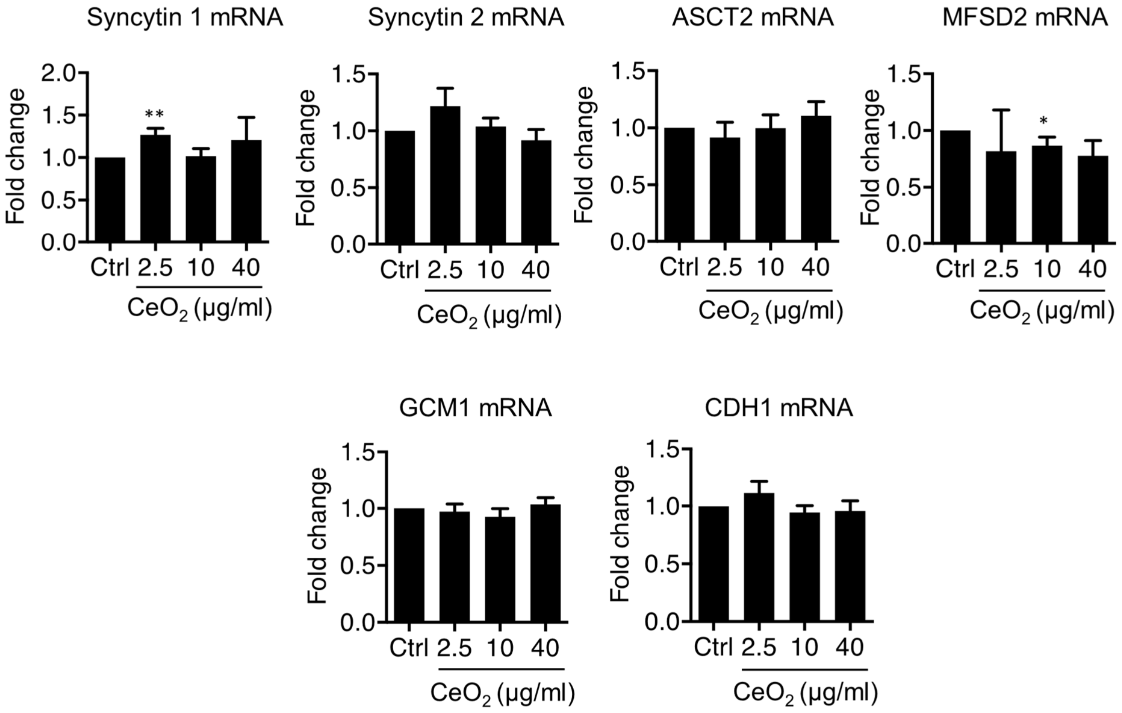

Figure S3. Effect of cerium dioxide nanoparticles on gene expression of trophoblast differentiation markers. VCT purified from term placenta were plated overnight and then were either left untreated (Ctrl) or incubated with CeO2 NPs at the indicated concentrations for 24 h. Total mRNA was extracted, reverse transcribed and transcript levels of syncytin-1, syncytin-2, ASCT2, MFSD2, GCM1 and CDH1 were determined by qPCR and represented as fold change means +/- SEM of n = 3.

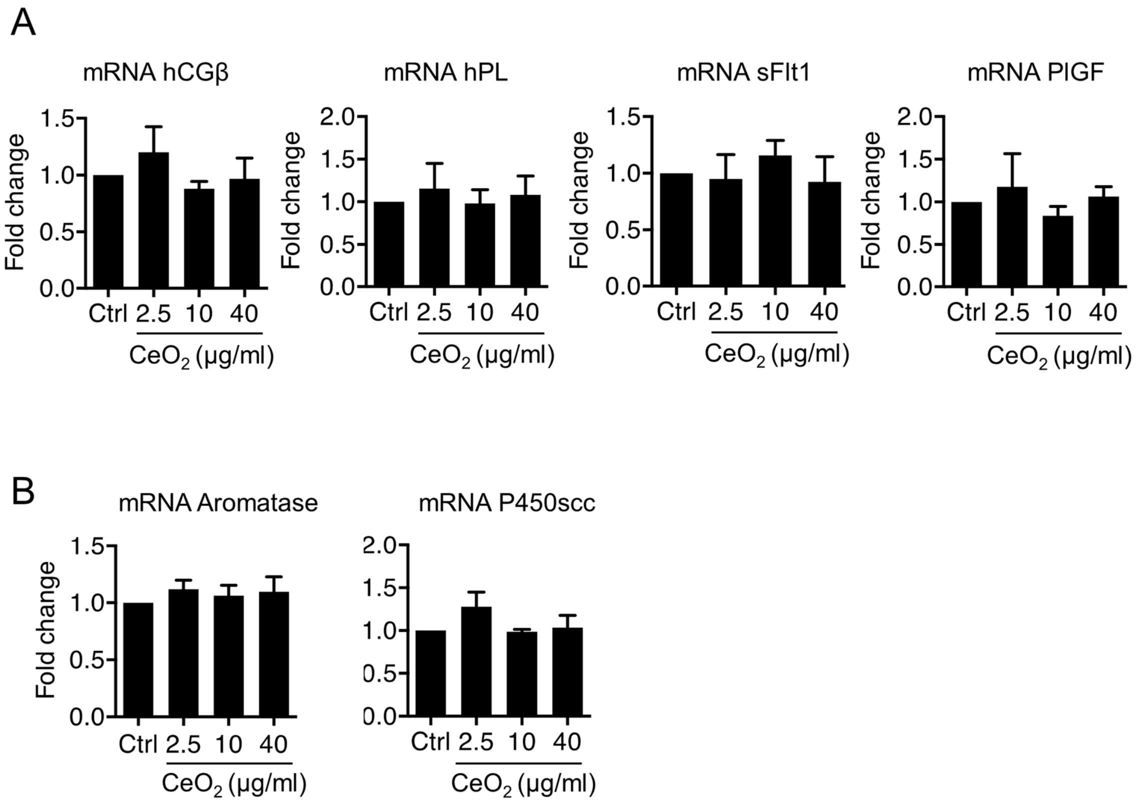

Figure S**4.** Effect of cerium dioxide nanoparticles on gene expression of actors involved in endocrine functions. VCT purified from term placenta were plated overnight and then were either left untreated (Ctrl) or incubated with CeO_2 NPs at the indicated concentrations for 24 h. Total mRNA was extracted and reverse transcribed. (**A**) The transcript levels of placental hormone hCGβ, hPL, sFlt1 and PlGF were determined by qPCR and represented as fold change means +/- SEM of n = 3. (**B**) The levels of two enzymes involved in trophoblast steroidogenesis, aromatase and P450scc, were determined by qPCR and represented as fold change means +/- SEM of n = 3.
